# ZIP4 upregulation aggravates nucleus pulposus cell degradation by promoting inflammation and oxidative stress by mediating the HDAC4-FoxO3a axis

**DOI:** 10.18632/aging.205412

**Published:** 2024-01-12

**Authors:** Mingkui Shen, Kuankuan Li, Lulu Wang, Li Feng, Xinyu Zhang, Haoping Zhang, Honggang Zhou, Guoxian Pei

**Affiliations:** 1 Intervertebral Disc Center, Third Hospital of Henan Province, Zhengzhou 450006, Henan, China; 2 School of Medicine, Southern University of Science and Technology, Shenzhen 518055, China

**Keywords:** ZIP4, nucleus pulposus cells, extracellular matrix degradation, HDAC4, FoxO3a

## Abstract

Background: Extracellular matrix metabolism dysregulation in nucleus pulposus (NP) cells represents a crucial pathophysiological feature of intervertebral disc degeneration (IDD). Our study elucidates the role and mechanism of Testis expressed 11 (TEX11, also called ZIP4) extracellular matrix degradation in the NP.

Materials and methods: Interleukin-1β (IL-1β) and H_2_O_2_ were used to treat NP cells to establish an IDD cell model. Normal NP tissues and NP tissues from IDD patients were harvested. ZIP4 mRNA and protein profiles in NP cells and tissues were examined. Enzyme-linked immunosorbent assay (ELISA) confirmed the profiles of TNF-α, IL-6, MDA, and SOD in NP cells. The alterations of reactive oxygen species (ROS), lactate dehydrogenase (LDH), COX2, iNOS, MMP-3, MMP-13, collagen II, aggrecan, FoxO3a, histone deacetylase 4 (HDAC4), Sirt1 and NF-κB levels in NP cells were determined using different assays.

Results: The ZIP4 profile increased in the NP tissues of IDD patients and IL-1β- or H_2_O_2_-treated NP cells. ZIP4 upregulation bolstered inflammation and oxidative stress in NP cells undergoing IL-1β treatment and exacerbated their extracellular matrix degradation, whereas ZIP4 knockdown produced the opposite outcome. Mechanistically, ZIP4 upregulated HDAC4 and enhanced NF-κB phosphorylation while repressing Sirt1 and FoxO3a phosphorylation levels. HDAC4 knockdown or Sirt1 promotion attenuated the effects mediated by ZIP4 overexpression in NP cells.

Conclusions: ZIP4 upregulation aggravates the extracellular matrix (ECM) degradation of NP cells by mediating inflammation and oxidative stress through the HDAC4-FoxO3a axis.

## INTRODUCTION

Low back pain (LBP) is a significant clinical symptom that accompanies nearly 80% of the population and it remains one of the leading causes of disability-adjusted life-years (DALYs) worldwide [1, 2]. Notably, intervertebral disc degeneration (IDD) has been regarded as a vital contributor to LBP, accounting for 26–42% of patients with LBP [3]. IDD arises from multiple factors, such as inflammation, oxidative stress, and mechanical stress. These factors facilitate the decline of nucleus pulposus (NP) progenitor cells, thus culminating in intervertebral disc dysfunction and structural destruction [4]. IDD has a sophisticated pathogenesis that has not been completely investigated. Current therapies aiming at reducing or controlling pain do not reverse the process of IDD [2]. Therefore, we need to delve into the pathogenesis of IDD to discover novel underlying treatment options.

The disruption of extracellular matrix (ECM) synthesis and degradation equilibrium plays a contributory role in instigating the disturbance observed in matrix components. Dysregulated expression and activation of matrix metalloproteinases (MMPs) are significant factors in ECM degradation [5]. MMPs, a protease family that depends on zinc and calcium ions, extensively participate in degrading all kinds of ECM matrices in the body [6]. Zinc ions are important constituents of metalloproteinases. Changes in the concentration of intracellular zinc ions pertain to IDD [7]. Zinc homeostasis is indispensable for sustaining normal cellular functions [8]. The zinc transporter family, one of the molecular mechanisms of zinc homeostasis, exerts a significant function in regulating the dynamic equilibrium of zinc ions [9]. ZIP4 belongs to the SLC39A family of zinc transporters, and exhibits altered levels in numerous types of cancer, such as ovarian cancer [10] and non-small cell lung cancer [11]. It facilitates cancer cell proliferation, migration, invasion, and metastasis. Notwithstanding, how ZIP4 functions under IDD circumstances remains poorly understood.

Histone deacetylase 4 (HDAC4), belonging to the HDAC family, functions importantly in transcriptional regulation and cell cycle development [12]. Reportedly, HDAC4 expression is upregulated in the intervertebral disc tissues of IDD mice, while HDAC4 overexpression bolsters NP cell apoptosis and exacerbates IDD in mice [13]. Engaging in a plethora of biological activities, including cell proliferation, apoptosis, oxidative stress, and inflammation [14], FoxO3a, a constituent of the forkhead transcription factor family, demonstrates its multiple functions, such as proliferation, apoptosis, cell cycle regulation, and DNA damage [15]. Enhanced FoxO3a fosters NP cell proliferation and suppresses NP cell apoptosis, hence mitigating IDD [16]. FoxO3a is known as a downstream molecule of HDAC4. Its functional mechanism in IDD still needs more investigation.

In this investigation, a noteworthy elevation in the profile of ZIP4 was discovered in both NP tissues of IDD patients and NP cells following IL-1β treatment. Through ZIP4 overexpression, facilitation of oxidative stress, inflammation, apoptosis, and ECM degradation was observed in NP cells treated with IL-1β. Regarding the mechanism, ZIP4 downregulated the HDAC4-FoxO3a pathway; HDAC4 overexpression abated the damage-boosting function of ZIP4 overexpression in the *ex vivo* IDD model; FoxO3a knockdown offset the damage-promoting function of ZIP4 in the *in vitro* IDD model. We hypothesized that ZIP4 upregulation regulated the HDAC4-FoxO3a axis to expedite IDD progression.

## MATERIALS AND METHODS

### Clinical samples

We harvested NP tissues from 30 people suffering from IDD (51-71 years of age) and normal NP tissues from 30 patients with spinal cord damage (32-49 years of age) from Third Hospital of Henan Province. The tissues were kept in liquid nitrogen at a temperature of -80° C. Our research, conducted under the approval of Third Hospital of Henan Province, adhered to the ethical guidelines outlined in the Declaration of Helsinki. Prior to participation, all individuals provided their informed consent by signing the appropriate documentation. The degree of IDD was evaluated by Pfirrmann grades according to T2-weighted section images.

### Culture and treatment of cells

Human NP cells were acquired from ScienCell Research Laboratories (Carlsbad, CA, USA). and cultivated in NP cell culture medium (ScienCell Research Laboratories). The cells were incubated in a humidified environment (37° C, 5% CO_2_). NP cells were first separated from the nucleus pulposus of human intervertebral discs. An IDD cell model was established by treating NP cells with IL-1β (5, 10, 20, 50 ng/ml) [17] or H_2_O_2_ (10, 25, 50, 100 μM) [18] for 24 hours. IL-1β (Order No. C600002) was purchased from Sangon Biotech (Shanghai, China) and H_2_O_2_ (Order No. 1.08600) was produced by Sigma-Aldrich (St. Louis, MO, USA). The Sirt1 activator resveratrol (Resv, Cat. HY-16561, MedChemExpress, Monmouth Junction, NJ, USA) was used to activate Sirt1 in NP cells at a dose of 30 μM [19].

### Cell transfection

Shanghai GeneChem Co., Ltd. provided us with ZIP4 overexpression plasmids (5 μg/ml), si-HDAC4 (50 nM), si-ZIP4 (50 nM), and their corresponding negative controls. Lipofectamine 2000 reagent (Invitrogen, Thermo Fisher Scientific, Waltham, MA, USA), NP cells were transfected after being seeded onto 6-well plates at a density of 1×10^5^ cells/mL. Twenty-four hours later, western blotting was used to evaluate the transfection efficiency.

### Cell viability detection

The CCK-8 (Cell Counting Kit-8) assay was used method to assess cell viability using a CCK8 kit (Cat.NO. CC1410-100, G-CLONE, Beijing, China). Briefly, NP cells were into 96-well plates (5,000 cells per well). After 24 hours for cell adhering and growing, the cells were treated with different experimental conditions for 24 hours. The CCK-8 reagent was diluted in the appropriate culture medium according to the manufacturer’s instructions, then the culture medium was replaced with the CCK-8 reagent. After incubating the cells for 2 hours, the absorbance of the samples was measured using a microplate reader at a wavelength of 450 nm.

### Detection of reactive oxygen species (ROS) levels

In accordance with the supplier’s guidelines, the measurement of ROS levels in NP cells was performed using 2ʹ,7ʹ-dichlorodihydrofluorescein diacetate (DCFH-DA) staining (Beyotime, Shanghai, China). After the designated treatment, cells in each experimental group were subjected to a 30-minute incubation in a completely dark environment at a temperature of 37° C with 5 μM DCFH-DA (Order No. 35845, Sigma-Aldrich, St. Louis, MO, USA). A fluorescence microscope was exploited to monitor the fluorescence intensity.

### Enzyme-linked immunosorbent assay (ELISA)

The conditioned medium of NP cells in each group was harvested, followed by 15 minutes of centrifugation (5000 rpm) at 4° C. The supernatant was kept at -80° C in preparation for the following experiments. ELISA kits (Westang, Shanghai, China) were used to confirm the levels of TNF-α (Order No. F02810) and IL-6 (Order No. F01310), both inflammatory factors, in the supernatant of NP cells.

### Lactate dehydrogenase (LDH) and oxidative stress mediator detection

The LDH Cytotoxicity Assay Kit (Cat. No. C0016, Beyotime, China) gauged the LDH level in NP cells undergoing IL-1β treatment in keeping with the instructions of the manufacturer. Commercial kits, specifically MDA (Cat. No. A003-1-2) and SOD (Cat. No. A001-3-2) acquired from Nanjing Jiancheng Bioengineering Institute (Jiangsu, China) were employed to assess the levels of oxidative stress mediators (MDA, SOD) in NP cells. These steps were meticulously carried out as per the instructions provided by the manufacturers.

### Western blot

To extract total protein from NP cells, a total protein extraction kit (Beyotime Biotechnology, Shanghai, China) was used. To ascertain the protein concentration, a bicinchoninic acid (BCA) protein analysis kit (Beyotime Biotechnology, Shanghai, China) was used. Subsequently, the protein samples were subjected to polyacrylamide gel electrophoresis (PAGE) using sodium dodecyl sulfate (SDS) and later transferred onto polyvinylidene difluoride (PVDF) membranes (Millipore, Bedford, MA, USA). With 5% skimmed milk adopted for 1-2 hours of sealing, the membranes underwent overnight incubation at a temperature of 4° C with the following primary antibodies: Anti-ZIP4 (Thermo Fisher Scientific, USA 1:1000, PA5-101971), Anti-COX2 (Abcam, Shanghai, China 1:1000, ab62331), Anti-iNOS (Abcam, 1:1000, ab178945), Anti-MMP-3 (Abcam, 1:1000, ab52915), Anti-MMP-13 (Abcam, 1:1000, ab39012), Anti-collagen II (Abcam, 1:1000, ab188570), Anti-aggrecan (Abcam, 1:1000, ab3778), Anti-HDAC4 (Abcam, 1:1000, ab12172), Anti- FoxO3a (phospho S253) (Abcam, 1:1000, ab154786), Anti-FoxO3a (Cell Signaling Technology, 1:500, #12829), Anti-Sirt1 (Abcam, 1:1000, ab110304), Anti-NF-kB p65 (phospho S536) (Abcam, 1:1000, ab86299), Anti-NF-κB (Abcam, 1:1000, ab32536), and Anti-GAPDH (Abcam, 1:1000, ab9485). The membranes underwent a thorough washing process using Tris-buffered saline with Tween-20 (TBST) on three occasions, followed by incubation with the appropriate secondary antibody (Cell Signaling Technology, Danvers, MA, USA) at a temperature of 37° C for one hour. To facilitate color development and image visualization, we harnessed enhanced chemiluminescence (ECL) western blotting substrate (Thermo Fisher Scientific, USA)

### Reverse transcriptase polymerase chain reaction (RT-PCR)

To extract the total RNA from NP cells, TRIzol reagent (Invitrogen; Thermo Fisher Scientific, USA) was utilized. For the subsequent reverse transcription step, Prime Script™ RT Master Mix (TaKaRa Bio) was introduced to convert the RNA into complementary DNA (cDNA). For RT-PCR analysis, iTaq™ Universal One-Step iTaq™ Universal SYBR^®^ Green Supermix (Bio-Rad Laboratories, Hercules, CA, USA) was applied in conjunction with the ABI 7500 instrument (Applied Biosystems; Thermo Fisher Scientific, USA). The internal control, glyceraldehyde-phosphate dehydrogenase (GAPDH), was utilized, and the relative profile of ZIP4 was computed via the 2^-∆∆CT^ approach. Below is detailed information on the primer sequences: ZIP4 forward 5’-ATGTCAGGAGCGGGTCTTGC-3’, reverse 5’-GCTGCTGTGCTGCTGGAAC-3’; MMP-3 forward 5’- GCAGTTTGCTCAGCCTATCC -3’, reverse 5’- GAGTGTCGGAGTCCAGCTTC -3’; MMP-13 forward 5’- TTGAGCTGGACTCATTGTCG -3’, reverse 5’- GGAGCCTCTCAGTCATGGAG -3’; collagen-II forward 5’- AGCTAAGCCCTGGGAAGAAG -3’, reverse 5’- AGGAGGTCCTTTGGGTCCTA -3’; aggrecan forward 5’- ACAGCTGGGGACATTAGTGG -3’, reverse 5’- GTGGAATGCAGAGGTGGTTT -3’; GAPDH forward 5’-ACAACTTTGGTATCGTGGAAGG-3’, reverse 5’-GCCATCACGCCACAGTTTC-3’.

### Analysis of statistics

All experimental procedures were replicated three times to ensure the accuracy and reliability of the outcomes. GraphPad Prism 8.0 software (GraphPad Inc., San Diego, CA, USA) was used for data analysis. The data are displayed as the mean ± standard deviation (SD). To compare two groups, an independent sample t test was implemented, whereas for comparisons among multiple groups, one-way analysis of variance (ANOVA) was utilized. A statistical significance level of *P*<0.05 was considered indicative of significant differences.

### Availability of data

The datasets during current study are available from the corresponding author on reasonable request.

## RESULTS

### The ZIP4 profile increased in IDD NP cell models

**Figure 1:**
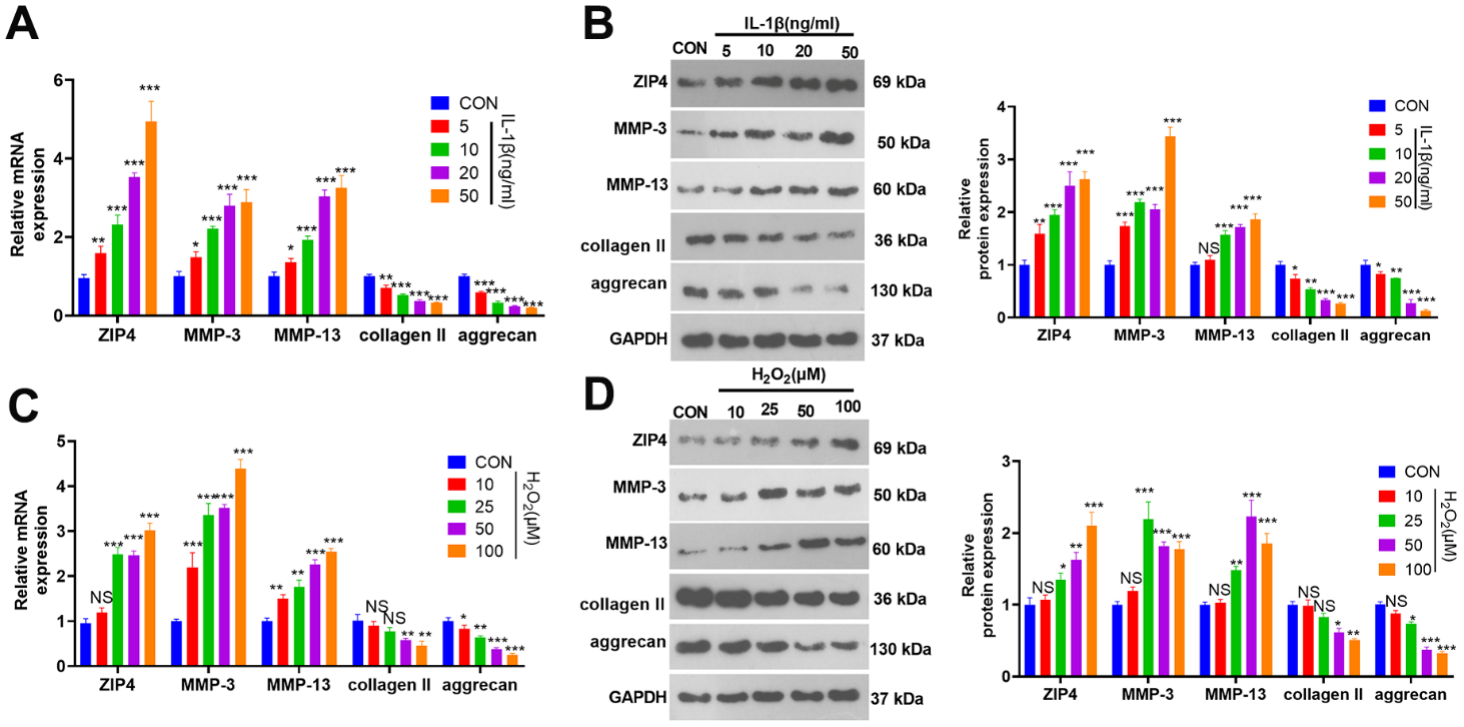
**ZIP4 expression changes in NP cells after treatment with IL-1β or H_2_O_2_.** (**A**, **B**) NP cells were treated with IL-1β (5, 10, 20, 50 ng/ml) for 24 hours. RT-PCR and western blotting were used to check ZIP4, MMP3, MMP13, collagen II, and aggrecan mRNA and protein profiles in NP cells. (**C**, **D**) NP cells were treated with H_2_O_2_ (10, 25, 50, and 100 μM) for 24 hours. RT-PCR and western blotting were used to determine ZIP4, MMP3, MMP13, collagen II, and aggrecan mRNA and protein profiles in NP cells. N=3. *P<0.05, **P<0.01, ***P<0.001 (vs. CON).

In an effort to investigate the expression characteristics of ZIP4 in the *ex vivo* IDD model, we exposed NP cells to varying concentrations of IL-1β (5, 10, 20, and 50 ng/ml) for 24 hours. RT-PCR and western blot analyses indicated that, compared to the CON group, ZIP4, MMP-3, and MMP-13 levels in NP cells exhibited a concentration-dependent increase in response to IL-1β treatment, while collagen II and aggrecan levels were repressed (Figure 1A, 1B). Following the preceding experiment, NP cells were subjected to various concentrations of H_2_O_2_ (10, 25, 50, 100 μM). RT-PCR and western blot analyses revealed that, when compared to the CON group, H_2_O_2_ treatment led to an augmentation in the profiles of ZIP4, MMP-3, and MMP-13 in NP cells while concurrently reducing collagen II and aggrecan levels (Figure 1C, 1D). These phenomena demonstrated that ZIP4 level is increased in NP cells treated with IL-1β and H_2_O_2_.

### ZIP4 level is increased in the NP tissues of IDD patients

**Figure 2:**
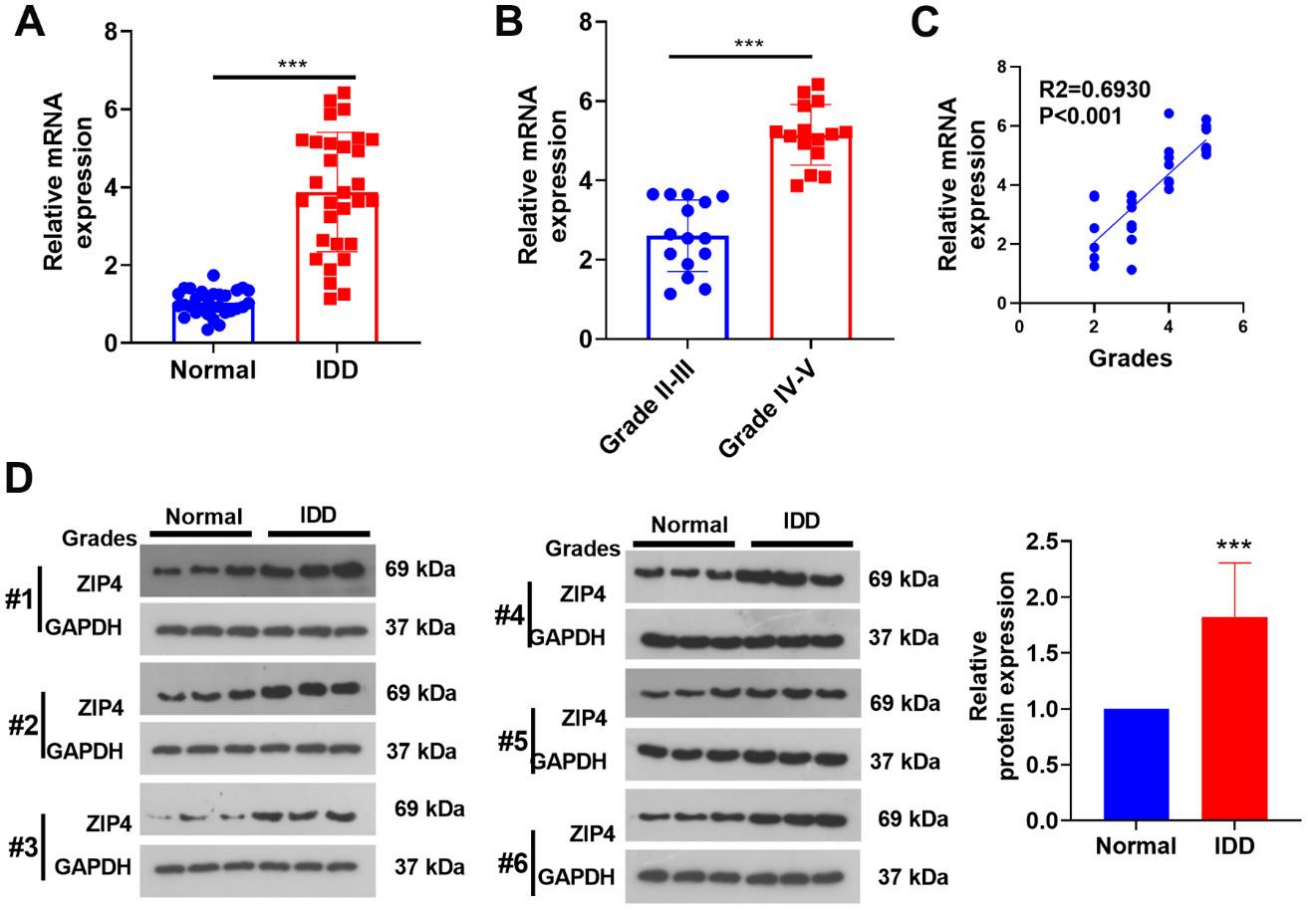
**ZIP4 expression in the NP tissues of IDD patients.** The NP tissues of IDD patients and normal patients were collected. (**A**) RT-PCR was used to ascertain the ZIP4 mRNA profile. (**B**) ZIP4 mRNA levels in different grades of IDD patients. (**C**) Correlation between ZIP4 mRNA level and grades of IDD patients. (**D**) Western blotting was used to detect ZIP4 protein level in the NP tissues of both IDD and normal patients. N=3. ***P<0.001.

In this investigation, NP tissues from both IDD patients and normal patients were harvested. The mRNA profile of ZIP4 was subsequently analyzed using RT-PCR. Based on the obtained data, a notable increase in the ZIP4 mRNA level was observed in the IDD patient NP tissues compared to the non-IDD patient NP tissues (Figure 2A). In addition, the ZIP4 mRNA level was promoted in grade III-IV IDD patients (vs. grade I-II IDD patients, Figure 2B). In addition, ZIP4 level had a positive relationship with the grades of IDD patients (Figure 2C). Through western blot analysis, it was revealed that the protein expression of ZIP4 exhibited a remarkable augmentation in the nucleus pulposus (NP) tissues obtained from patients diagnosed with IDD, in stark contrast to the protein level observed in the NP tissues of normal patients (Figure 2D).

### ZIP4 upregulation aggravates ECM degradation in NP cells following IL-1β treatment

**Figure 3:**
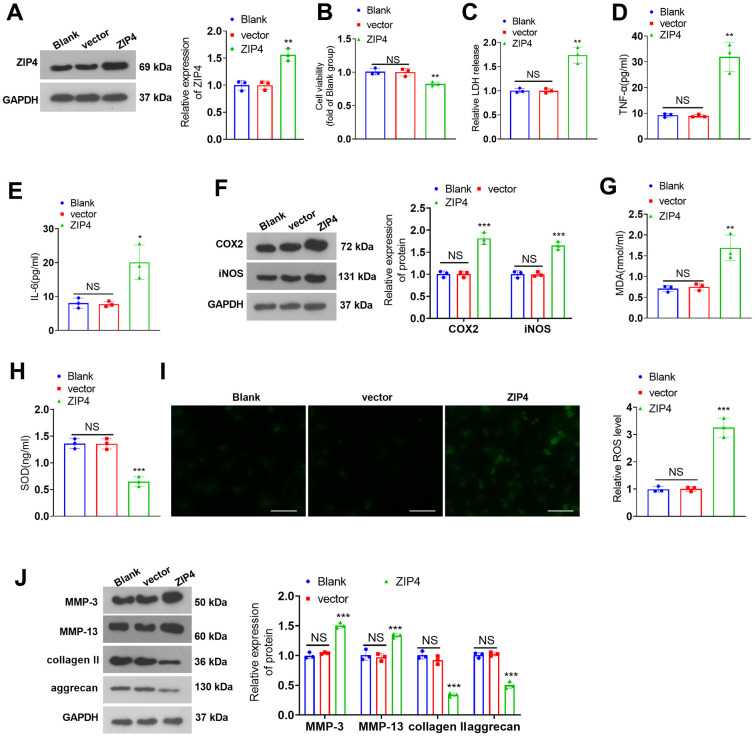
**ZIP4 upregulation aggravates ECM degradation in NP cells.** (**A**) NP cells transfected with the vector and ZIP4 overexpression plasmids. Western blot experiments were used to check the transfection efficiency. (**B**) CCK8 assay was used for evaluating cell viability. (**C**) The cytotoxicity detection kit evaluated LDH release in NP cells following IL-1β treatment. (**D**, **E**) TNF-α and IL-6 profiles in the cells verified by ELISA. (**F**) Western blot showing the expression levels of COX2 and iNOS in the cells. (**G**–**I**) MDA, SOD, and ROS levels in the cells were determined. Scale bar=100 μm. (**J**) MMP-3, MMP-13, collagen II and aggrecan levels in the cells confirmed by western blot. N=3. NS P>0.05, *P<0.05, **P<0.01, ***P<0.001 (vs. vector).

**Figure 4:**
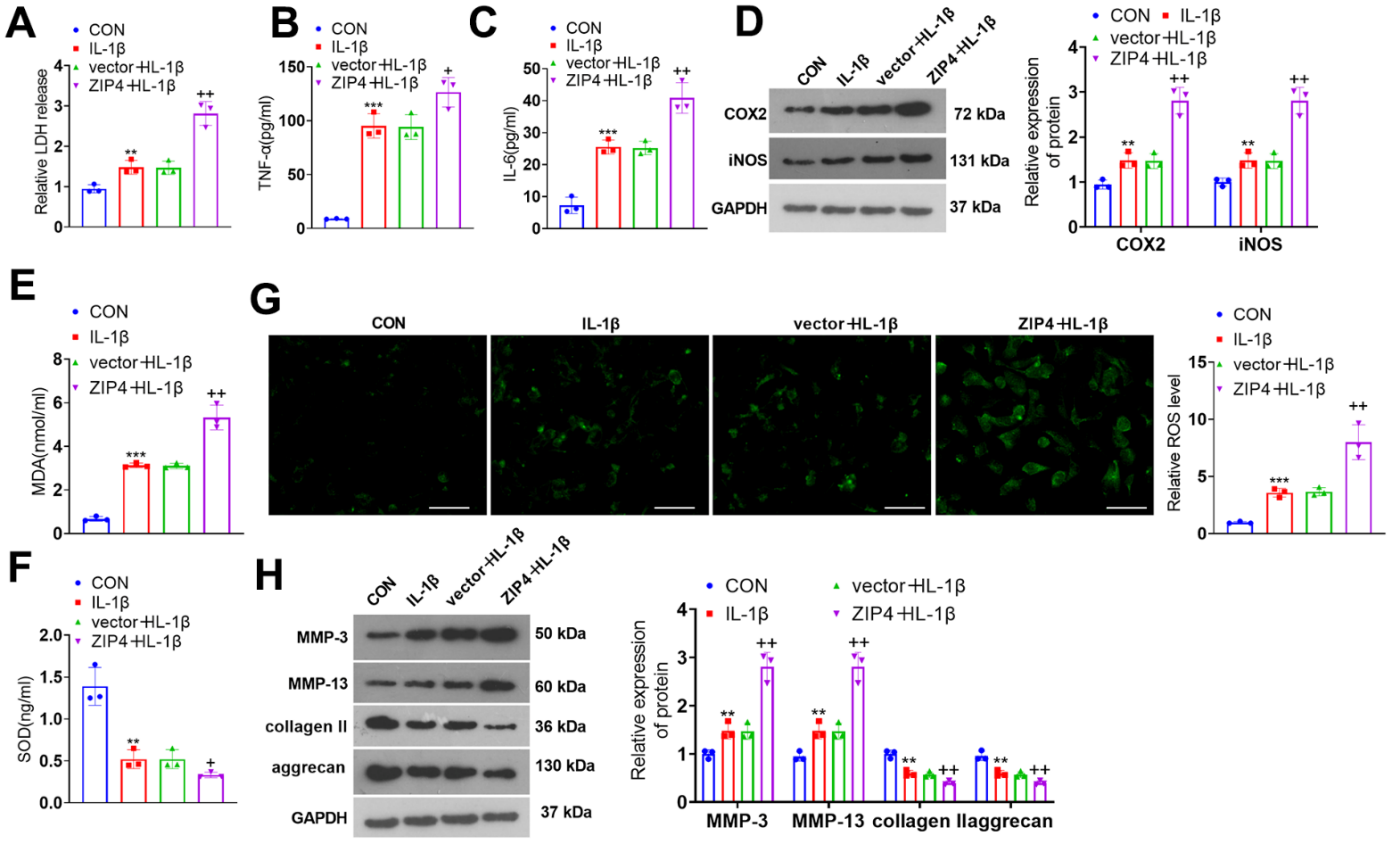
**ZIP4 upregulation aggravates ECM degradation in NP cells undergoing IL-1β treatment.** NP cells transfected with the vector and ZIP4 overexpression plasmids were subjected to IL-1β (20 ng/ml) treatment for a 24-hour period. (**A**) The cytotoxicity detection kit evaluated LDH release in NP cells following IL-1β treatment. (**B**, **C**) TNF-α and IL-6 profiles in the cells verified by ELISA. (**D**) Western blot showing the expression levels of COX2 and iNOS in the cells. (**E**–**G**) MDA, SOD, and ROS levels in the cells. Scale bar=100 μm. (**H**) MMP-3, MMP-13, collagen II and aggrecan levels in the cells confirmed by western blot. N=3. **P<0.01, ***P<0.001 (vs. CON); *+P<0.05, ++P<0.01* (vs. vector+IL-1β).

To investigate the role of ZIP4 in IDD, a ZIP4 overexpression cell model was constructed (Figure 3A). Cell viability was determined and the result showed that ZIP4 overexpression reduced cell viability (Figure 3B). In addition, ZIP4 overexpression promoted LDH, TNF-α and IL-6 production (Figure 3C–3E). Further experiments showed that the levels of COX2, iNOS, MDA and ROS were elevated after ZIP4 upregulation, while SOD level was reduced (Figure 3F–3I). Western blot revealed that following ZIP4 upregualtion, ECM of NP cells were enhanced (Figure 3J). Next, NP cells were treated with IL-1β (20 ng/ml) following transfection for 24 hours. Compared to the CON group, IL-1β expanded LDH release in NP cells. When matched against the vector+IL-1β group, ZIP4 overexpression augmented LDH release in NP cells undergoing IL-1β treatment (Figure 4A). ELISA showed that vis-à-vis CON, an increase was discovered in TNF-α and IL-6 profiles in NP cells subjected to IL-1β treatment. As opposed to the vector+IL-1β group, ZIP4 overexpression boosted the profiles of the inflammatory factors in NP cells subsequent to IL-1β treatment (Figure 4B, 4C). ZIP4 overexpression increased COX2 and iNOS expression in the cells (Figure 4D). Moreover, ZIP4 overexpression upregulated MDA and ROS levels and downregulated SOD levels in the cells (Figure 4E–4G). Western blot showed that the levels of MMP-3 and MMP-13 were significantly elevated in NP cells treated with IL-1β, and further elevated after ZIP4 overexpression. By contrast, collagen II and aggrecan were noticeably reduced in the NP cells undergoing IL-1β treatment and further repressed after ZIP4 overexpression (Figure 4H). These findings confirmed that ZIP4 upregulation fostered inflammation, oxidative stress, and apoptosis in NP cells and exacerbated their ECM degradation.

### ZIP4 knockdown alleviates ECM degradation in NP cells treated with IL-1β

**Figure 5:**
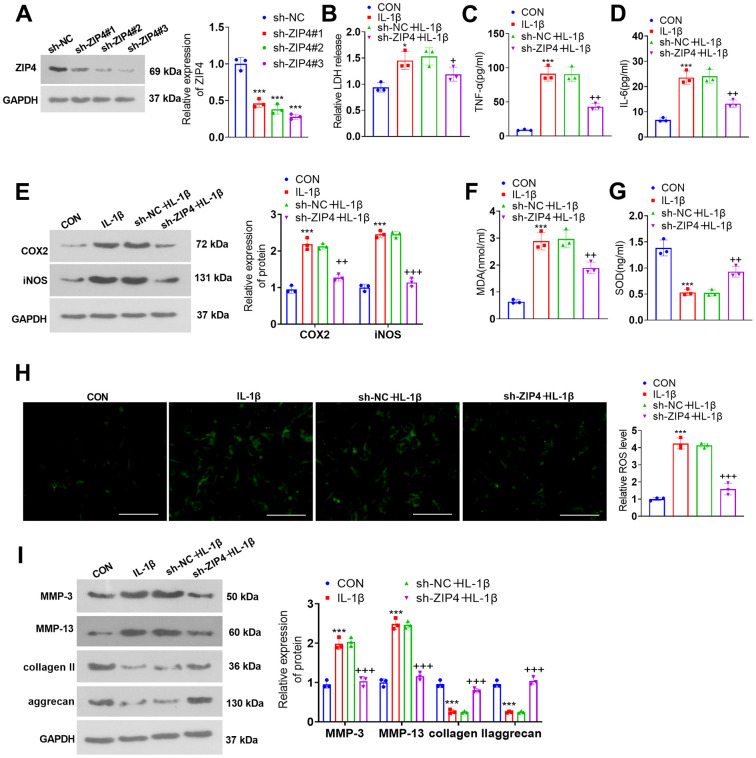
**ZIP4 knockdown mitigates ECM degradation in NP cells treated with IL-1β.** (**A**) NP cells were transfected with sh-NC and sh-ZIP4. Western blot analysis confirmed the profile of ZIP4 in transfected NP cells. Subsequent to transfection, the cells underwent IL-1β (20 ng/ml) treatment for a duration of 24 hours. (**B**) LDH release in NP cells was evaluated. (**C**, **D**) ELISA was used to determine TNF-α and IL-6 expression in the cells. (**E**) Western blot verified COX2 and iNOS expression in the cells. (**F**–**H**) Alterations in MDA, SOD, and ROS levels in NP cells were detected. Scale bar=100 μm. (**I**) MMP-3, MMP-13, collagen II, and aggrecan profiles in the cells checked by western blot. N=3. *P<0.05, ***P<0.001 (vs. CON); *+P<0.05, ++P<0.01, +++P<0.001* (vs. sh-NC+IL-1β).

To delve into the function of ZIP4 in IDD, we transfected NP cells with sh-NC and sh-ZIP4 (Figure 5A). ZIP4 knockdown suppressed LDH, TNF-α and IL-6 profiles in NP cells exposed to IL-1β (Figure 5B–5D). The western blot results indicated that in relation to the sh-NC+IL-1β group, ZIP4 knockdown decreased COX2 and iNOS levels in the NP cells (Figure 4E). ZIP4 knockdown lowered MDA and ROS levels and increased SOD levels in NP cells treated with IL-1β (versus sh-NC+IL-1β) (Figure 5F–5H). As suggested by western blot, compared to the sh-NC+IL-1β group, ZIP4 knockdown repressed MMP-3 and MMP-13 expression and boosted collagen II and aggrecan expression in the treated cells (Figure 5I). These phenomena confirmed that ZIP4 knockdown impeded NP cell inflammation, oxidative stress, and apoptosis and ameliorated ECM degradation in NP cells treated with IL-1β.

### The influence of ZIP4 on the HDAC4-FoxO3a pathway

**Figure 6:**
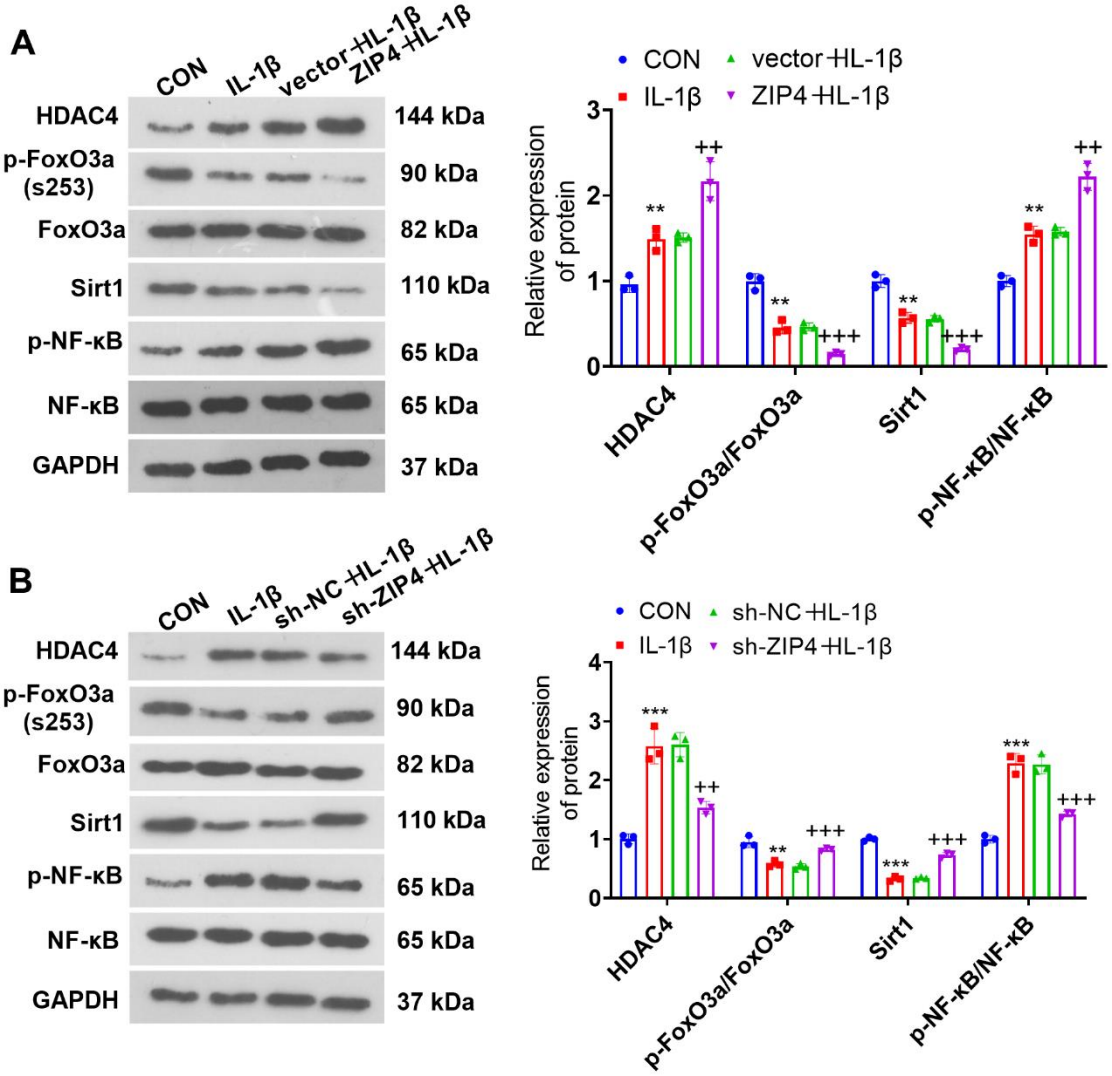
**The influence of ZIP4 on the HDAC4-FoxO3a pathway.** (**A**, **B**) Western blotting was implemented to determine the HDAC4, FoxO3a, Sirt1 and NF-κB profiles in NP cells after IL-1β treatment. N=3. **P<0.01, ***P<0.001 (vs. CON); *++P<0.01, +++P<0.001* (vs. vector+IL-1β or sh-NC+IL-1β).

To understand the impact of ZIP4 on the HDAC4/FoxO3a pathway, we gauged HDAC4, FoxO3a, Sirt1 and NF-κB levels in NP cells following IL-1β treatment subsequent to transfection via western blotting. In relation to CON, IL-1β promoted the profiles of HDAC4 and NF-κB phosphorylation while reducing FoxO3a phosphorylation and Sirt1 levels in NP cells (Figure 6A, 6B). In contrast to the corresponding control groups (vector+IL-1β or sh-NC+IL-1β), ZIP4 overexpression aggravated the elevation of HDAC4 and NF-κB phosphorylation and further reduced FoxO3a phosphorylation and Sirt1 levels in NP cells treated with IL-1β (Figure 6A). In contrast, ZIP4 knockdown reduced HDAC4 and NF-κB phosphorylation and significantly enhanced FoxO3a phosphorylation and Sirt1 levels in NP cells treated with IL-1β (Figure 6B). The above outcomes revealed that ZIP4 promoted the profile of HDAC4 and activated the Sirt1-NF-κB pathway.

### HDAC4 knockdown weakened the damage-boosting effects mediated by ZIP4 overexpression

**Figure 7:**
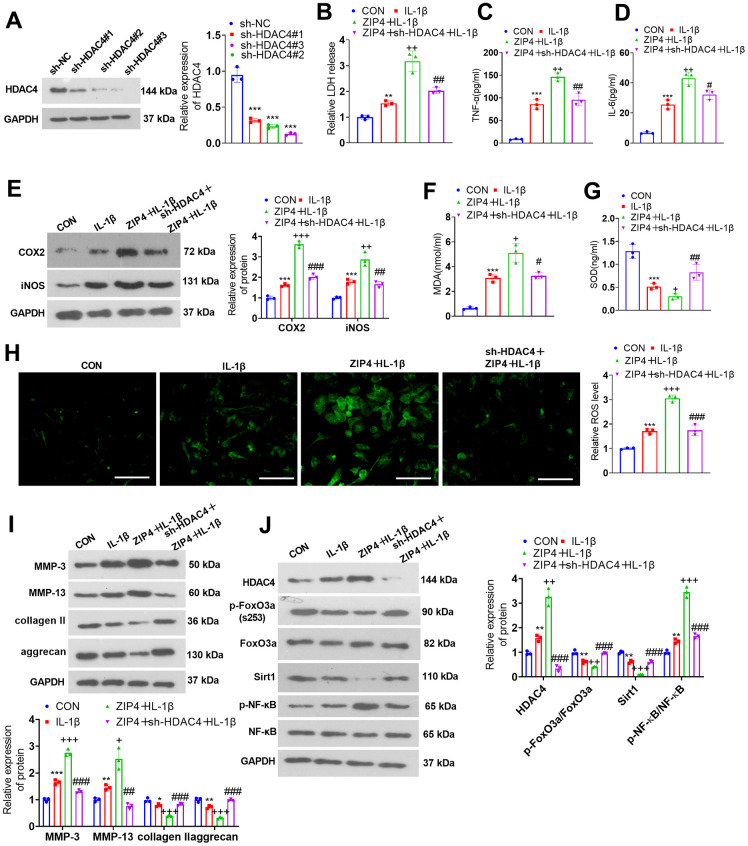
**HDAC4 overexpression abates the damage-boosting function mediated by ZIP4 overexpression.** (**A**) NP cells were transfected with the sh-NC or sh-HDAC4 overexpression plasmid. Western blot confirmed the transfection efficiency. The HDAC4 overexpression plasmid and ZIP4 overexpression plasmid were cotransfected into NP cells, which underwent 20 ng/ml IL-1β treatment for a 24-hour period in the following step. (**B**) The LDH level in NP cells treated with IL-1β. (**C**, **D**) The profiles of inflammatory factors in the cells verified by ELISA. (**E**) COX2 and iNOS profiles in the cells checked via western blot. (**F**–**H**) MDA, SOD, and ROS levels in the cells were detected. Scale bar=100 μm. (**I**) MMP-3, MMP-13, collagen II, and aggrecan profiles in the cells examined by western blot. (**J**) Western blot verified HDAC4, FoxO3a, Sirt1 and NF-κB profiles in NP cells undergoing IL-1β treatment. N=3. **P<0.01, ***P<0.001 (vs. CON); *+P<0.05, ++P<0.01, +++P<0.001* (vs. IL-1β); *#P<0.05, ##P<0.01, ###P<0.001* (vs. ZIP4+IL-1β).

For the purpose of probing the influence of HDAC4 on ZIP4, NP cells were transfected with sh-NC and sh-HDAC4. Western blotting was used to examine the transfection efficiency (Figure 7A). Next, NP cells were transfected with sh-HDAC4 and/or ZIP4 overexpression plasmids and then treated with 20 ng/ml IL-1β for 24 hours. Cytotoxicity examination demonstrated that compared with the ZIP4+IL-1β group, the sh-HDAC4+ZIP4+IL-1β group exhibited a decline in LDH release in NP cells (Figure 7B). Upon comparison to the ZIP4+IL-1β group, a notable reduction in TNF-α and IL-6 profiles was observed in NP cells within the sh-HDAC4+ZIP4+IL-1β group (Figure 7C, 7D). Western blot analysis suggested that in relation to the ZIP4+IL-1β group, sh-HDAC4 reduced the profiles of COX2 and iNOS in ZIP4-overexpressing NP cells treated with IL-1β (Figure 7E). MDA and ROS levels were lowered, while SOD levels were heightened in the sh-HDAC4+ZIP4+IL-1β group vis-à-vis the ZIP4+IL-1β group (Figure 7F–7H). Western blotting revealed that MMP-3 and MMP-13 expression was downregulated, while collagen II and aggrecan expression was upregulated in the sh-HDAC4+ZIP4+IL-1β group compared with the ZIP4+IL-1β group (Figure 7I). Western blot analysis suggested that the levels of HDAC4 and NF-κB were reduced, while FoxO3a phosphorylation and Sirt1 levels were enhanced in the sh-HDAC4+ZIP4+IL-1β group versus the ZIP4+IL-1β group (Figure 7J). These phenomena confirmed that HDAC4 knockdown weakened the damage-promoting function mediated by ZIP4 overexpression in the *in vitro* IDD model.

### FoxO3a knockdown offsets the damage-promoting function mediated by ZIP4 overexpression

**Figure 8:**
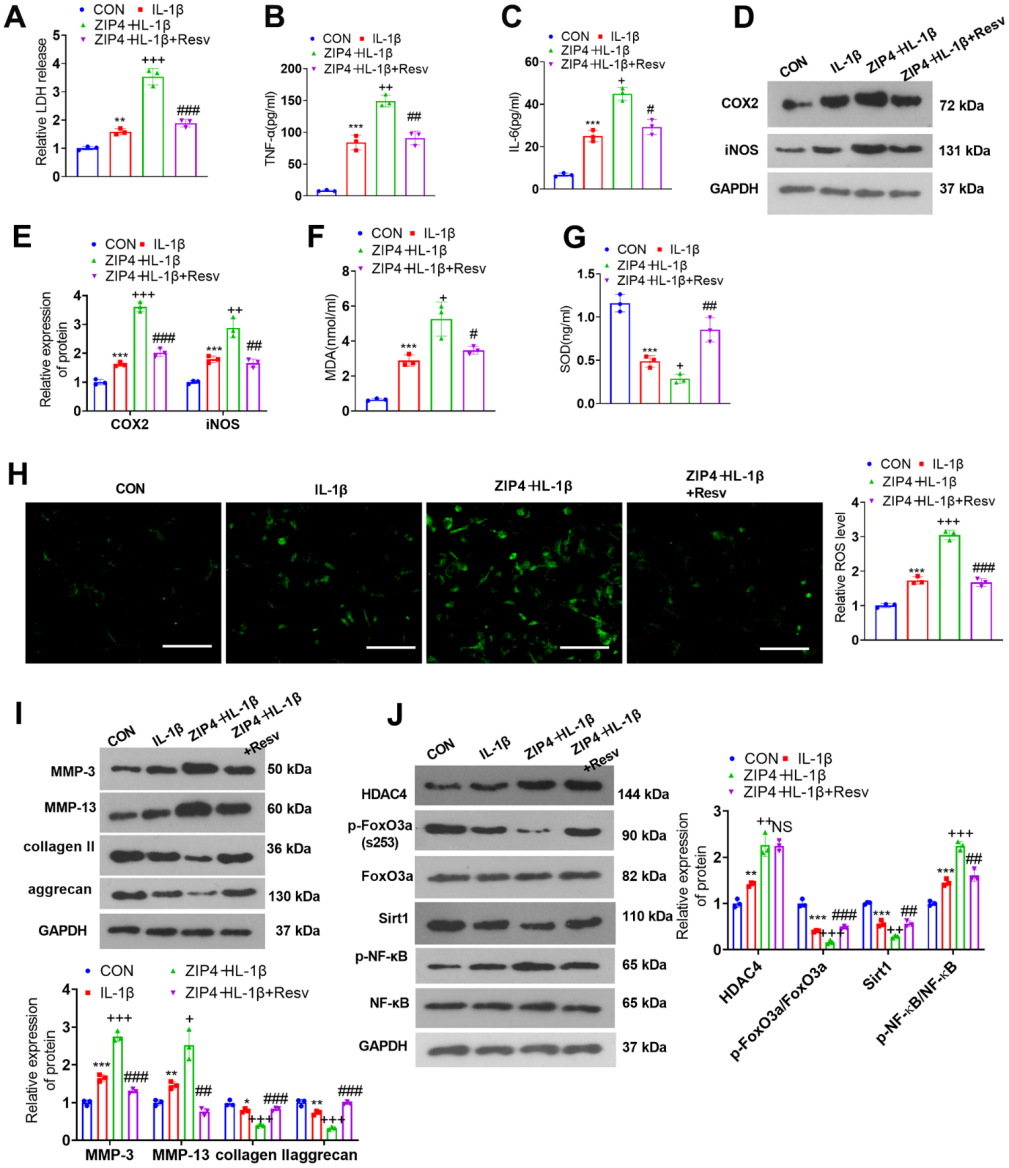
**FoxO3a knockdown offsets the damage-promoting function mediated by ZIP4 overexpression.** NP cells were treated with IL-1β (20 ng/ml) or Resv (30 μM) for 24 hours. (**A**) The LDH level in NP cells subjected to treatment with IL-1β. (**B**, **C**) The profiles of inflammatory factors in the cells checked by ELISA. (**D**, **E**) Western blot analysis confirmed COX2 and iNOS levels in the cells. (**F**–**H**) MDA, SOD, and ROS levels in the cells gauged through the assistance of commercial kits. Scale bar=100 μm. (**I**) MMP-3, MMP-13, collagen II, and aggrecan levels determined through western blot in the cells. (**J**) HDAC4, FoxO3a, Sirt1, and NF-κB profiles in NP cells following IL-1β treatment. N=3. *P<0.05, **P<0.01, ***P<0.001 (vs. CON); *+P<0.05, ++P<0.01, +++P<0.001* (vs. IL-1β); *#P<0.05, ##P<0.01, ###P<0.001* (vs. sh-FoxO3a+IL-1β).

To investigate the influence of Sirt1 on ZIP4-mediated effects, we treated NP cells with the Sirt1 activator Resv (30 μM). The cytotoxicity assay kit revealed that in contrast with the IL-1β group, Resv treatment reduced LDH release in ZIP4-overexpressing NP cells treated with IL-1β (Figure 8A). Based on the ELISA results, Resv treatment vigorously mitigated the expression levels of TNF-α and IL-6 in NP cells following IL-1β treatment when compared to the IL-1β group (Figure 8B, 8C). As suggested by western blot data, compared to the IL-1β group, Resv treatment repressed the profiles of COX2 and iNOS in NP cells following IL-1β treatment (Figure 8D, 8E). The levels of MDA and ROS were lowered after Resv treatment, and the level of SOD was enhanced (versus the ZIP4+IL-1β group, Figure 8F, 8H). Western blot confirmed that in contrast to the ZIP4+IL-1β group, Resv addition suppressed the profiles of MMP-3 and MMP-13 but enhanced those of collagen II and aggrecan in NP cells (Figure 8I). Western blot analysis revealed that Resv failed to influence HDAC4 expression but enhanced FoxO3a phosphorylation and Sirt1 expression in NP cells following IL-1β treatment. Meanwhile, the NF-κB phosphorylation level was significantly reduced by Resv treatment (Figure 8J). Therefore, Sirt1 activation repressed inflammation, oxidative stress, apoptosis, and ECM degradation in NP cells treated with IL-1β and offset the damage-boosting function mediated by ZIP4 overexpression in the *ex vivo* IDD model.

## DISCUSSION

IDD, a leading contributor to low back pain, neck pain, and relevant dysfunctions, lays the pathological foundation for intervertebral disc herniation, spinal stenosis, and other diseases [20]. The primary pathological alterations in IDD encompass fewer NP cells and ECM degradation [21]. Here, we discovered that ZIP4 expression was elevated in an *in vitro* IDD model and IDD tissues, whereas ZIP4 overexpression downregulated the HDAC4-FoxO3a pathway to facilitate inflammation and oxidative stress, hence exacerbating NP cell ECM degradation.

NP cells are indispensable for sustaining the function of the intervertebral disc. Early cell senescence and apoptosis are leading contributors to IDD, and the major manifestations include a reduction in the function and number of NP cells in degenerative intervertebral discs [22]. ECM degradation in the intervertebral disc can be attributed to the imbalance of ECM anabolism and catabolism, lessened ECM synthesis, and heightened activity of proteases that can degrade ECM, directly culminating in an increase in ECM catabolism [23]. Inflammatory factors exert a significant function in IDD progression. The interplay and aberrant expression of inflammatory factors can damage the balance of ECM decomposition and metabolism in the intervertebral disc, resulting in inflammatory responses and engaging in or boosting IDD development [24, 25]. IL-1β, a significant proinflammatory cytokine in the interleukin-1 (IL-1) family, is the primary cause of proteoglycan degradation in the intervertebral disc and an important factor for boosting high MMP expression. It is also a significant cytokine that induces IDD [26]. Moreover, H_2_O_2_ can also lead to oxidative stress, intervertebral disc cell death and ECM degradation [27]. Here, we established an *in vitro* IDD model by treating NP cells with IL-1β or H_2_O_2_. We uncovered that IL-1β bolstered TNF-α, IL-6, COX2, and iNOS expression in NP cells; MDA and ROS levels and MMP-3 and MMP-13 profiles dramatically increased in IL-1β- or H_2_O_2_-treated NP cells; and the profiles of SOD, collagen II, and aggrecan were dramatically decreased. Our findings revealed that IL-1β facilitated NP cell inflammation, oxidative stress, and ECM degradation.

Members of the zinc transporter family participate in IDD occurrence and development. For instance, zinc transporter ZIP8 (ZIP8) expression is notably heightened in denatured NP tissues, while ZIP8 downregulation hampers the profiles of ECM degrading enzymes and restores those of ECM proteins in NP cells undergoing treatment with IL-1β to retard IDD progression [28]. ZIP8 expression is remarkably elevated in NP cells subsequent to IL-1β treatment. ZIP8 knockdown alleviates the ECM degradation of NP cells elicited by inflammatory stimulation [29]. ZIP4 is a member of the zinc transporter SLC39A/ZIP family. Reportedly, ZIP4 overexpression suppresses hepatoma carcinoma cell apoptosis and bolsters their migration and invasion [30]. ZIP4 presents high expression in pancreatic cancer tissues. ZIP4 knockdown dampens pancreatic cancer cell migration and invasion, slowing pancreatic cancer development [31]. Here, we discovered that compared to the corresponding control group, the ZIP4 profile was elevated in NP cells following IL-1β treatment and IDD patient NP tissues. ZIP4 overexpression boosted inflammation, oxidative stress, and apoptosis in NP cells treated with IL-1β and exacerbated their ECM degradation, whereas ZIP4 knockdown reversed these effects. These discoveries confirmed that ZIP4, a pathogenic factor for IDD, can speed up IDD development.

Studies in recent years have suggested that HDAC4 exerts a regulatory function in endplate chondrocyte degeneration and NP cell degeneration [32]. For instance, HDAC4 bolsters morphological alterations in endplate chondrocytes and augments ECM degradation and endplate cartilage degeneration [33]. In an IDD mouse model, GSK3β was downregulated in intervertebral disc tissues, and upregulating GSK3β mitigated NP cell apoptosis and disc degeneration in IDD mice by repressing HDAC4 [13].

FoxO3a is a downstream molecule of HDAC4. HDAC4 can promote FoxO3a deacetylation to increase its transcriptional activity [34]. FoxO3a activation promotes superoxide dismutase 2 (SOD2) synthesis to cramp oxidative stress, hence effectively retarding IDD [35]. Here, we uncovered that ZIP4 upregulated HDAC4 and reduced FoxO3a phosphorylation. HDAC4 downregulation weakened the damage-promoting function mediated by ZIP4 overexpression in the *in vitro* IDD model, accompanied by FoxO3a phosphorylation upregulation. These outcomes demonstrated that ZIP4 modulated the HDAC4-FoxO3a axis to function in the context of IDD.

Sirt1, classified as a class III histone deacetylase, exhibits extensive involvement in the regulation of various age-related cellular mechanisms. These mechanisms encompass autophagy, apoptosis, energy metabolism, and antiaging processes [36]. During the progression of IVDD, Sirt1 is downregulated in degenerative discs and exerts a protective effect by regulating cellular senescence and promoting regeneration [37]. Interestingly, HDAC4 has an inhibitive effect on SIRT1 by inducing the loss of histone acetylation on the SIRT1 promoter region under proinflammatory cytokine interferon-gamma (IFN-γ) treatment [38]. In nasal epithelial cells, the HDAC4 profile was elevated upon IL-13 treatment, which repressed SIRT1 and initiated NF-κB signaling. HDAC4 knockdown activated SIRT1/NF-κB signaling and mitigated inflammatory responses and mucus generation in nasal epithelial cells after IL-13 treatment [39]. NF-κB, an indispensable transcription factor, plays a pivotal role in the modulation of IL-1β-elicited cell viability, migration, apoptosis, and inflammatory response in both human chondrocytes and NP cells [40–42]. SIRT1 overexpression reversed IL-1β-elicited ECM degradation and cell apoptosis by deacetylating RelA/p65 and inhibiting NF-κB nuclear translocation [43]. Here, we found that ZIP4 overexpression leads to reduced Sirt1 expression and enhanced NF-κB phosphorylation. HDAC4 knockdown enhanced Sirt1 while suppressing NF-κB pathway activation, and these results were consistent with the reduced inflammation and oxidative stress in NP cells. However, the administration of the Sirt1 activator Resv significantly improved IL-1β and ZIP4 overexpression-mediated apoptosis, inflammation, ECM degradation, and oxidative stress in NP cells. These data suggested that the ZIP4-HDAC4-Foxo3-Sirt1-NF-κB pathway plays a role in NP cell dysfunction.

However, several shortcomings need to be further investigated in the future. First, the functions of ZIP4 in mediating IDD progression should be confirmed in animals. Second, the upstream mechanism of ZIP4 in NP cells requires more experiments for clarification. Third, since cell death has a pivotal role in the dysfunctions of NP cells in IDD, further experiments should be conducted to determine cell apoptosis or autophagy after selectively regulating ZIP4 expression.

## CONCLUSIONS

To summarize, our research reveals that ZIP4 overexpression bolsters inflammation and oxidative stress in NP cells following IL-1β treatment and aggravates cell ECM degradation. Regarding the mechanism, ZIP4 downregulates HDAC4 and boosts FoxO3a acetylation. This study proposed that the novel ZIP4/HDAC4/FoxO3a/Sirt1/NF-κB axis exerts an important function in IDD. Our findings may afford new ways of thinking and novel targets for treating and ameliorating IDD.
